# New Cross-Talks between Pathways Involved in Grapevine Infection with ‘*Candidatus* Phytoplasma solani’ Revealed by Temporal Network Modelling

**DOI:** 10.3390/plants10040646

**Published:** 2021-03-29

**Authors:** Blaž Škrlj, Maruša Pompe Novak, Günter Brader, Barbara Anžič, Živa Ramšak, Kristina Gruden, Jan Kralj, Aleš Kladnik, Nada Lavrač, Thomas Roitsch, Marina Dermastia

**Affiliations:** 1 Jožef Stefan International Postgraduate School, 1000 Ljubljana, Slovenia; Nada.Lavrac@ijs.si; 2 Jožef Stefan Institute, 1000 Ljubljana, Slovenia; jan.kralj@ijs.si; 3 National Institute of Biology, 1000 Ljubljana, Slovenia; marusa.pompe.novak@nib.si (M.P.N.); barbara.anzic@gmail.com (B.A.); ziva.ramsak@nib.si (Ž.R.); kristina.gruden@nib.si (K.G.); marina.dermastia@nib.si (M.D.); 4 School of Viticulture and Enology, University of Nova Gorica, 5271 Vipava, Slovenia; 5 Austrian Institute of Technology, Bioresources Unit, 3430 Tulln, Austria; guenter.brader@ait.ac.at; 6 Department of Biology, Biotechnical Faculty, University of Ljubljana, 1000 Ljubljana, Slovenia; ales.kladnik@bf.uni-lj.si; 7 Department of Plant and Environmental Sciences, University of Copenhagen, 2630 Taastrup, Denmark; roitsch@plen.ku.dk

**Keywords:** network analysis, phytoplasma, bois noir, community detection, enrichment analysis

## Abstract

Understanding temporal biological phenomena is a challenging task that can be approached using network analysis. Here, we explored whether network reconstruction can be used to better understand the temporal dynamics of bois noir, which is associated with ‘*Candidatus* Phytoplasma solani’, and is one of the most widespread phytoplasma diseases of grapevine in Europe. We proposed a methodology that explores the temporal network dynamics at the community level, i.e., densely connected subnetworks. The methodology offers both insights into the functional dynamics via enrichment analysis at the community level, and analyses of the community dissipation, as a measure that accounts for community degradation. We validated this methodology with cases on experimental temporal expression data of uninfected grapevines and grapevines infected with ‘*Ca*. P. solani’. These data confirm some known gene communities involved in this infection. They also reveal several new gene communities and their potential regulatory networks that have not been linked to ‘*Ca*. P. solani’ to date. To confirm the capabilities of the proposed method, selected predictions were empirically evaluated.

## 1. Introduction

Bois noir (BN) is an important economic grapevine yellows disease that is caused by the phytopathogenic bacterium ‘*Candidatus* Phytoplasma solani’, from the solbur 16SrXII-A subgroup of the order Acholeplasmatales in the class Mollicutes [1]. This phytoplasma is endemic across a broad Mediterranean region [2,3,4,5,6], and it has also been reported from China, Chile and Canada [6]. Its spread occurs via a complicated disease cycle that includes insect vectors and multiple herbaceous plants as phytoplasma reservoirs [7,8]. In addition, different environmental conditions and grapevine cultivars also contribute to the development of BN disease [6]. Several studies of BN have shown transcriptional and metabolic changes in host plants [9,10,11,12,13,14,15,16] that involve the major plant signal transduction pathways. However, between these ‘highways’ of information flow, there are ‘side-streets’ that interconnect these pathways that are likely to be overlooked by classical methods, especially in a system as complex as BN. Some approaches to define how to combine these diverse data into a suitable model for explaining the pathogenicity of phytoplasmas that cause grapevine yellows have been proposed recently [17,18,19,20].

The analysis of RNA sequencing (RNA-Seq) data is becoming one of the prevailing empirical approaches to understanding a wide array of biological systems. In recent years, it has also become increasingly possible to perform sequencing across time, and thus to obtain multiple gene expression ‘snapshots’ of the same organism or its tissues that can be used for the more accurate analysis of time-dependent phenomena, such as the progression of disease. As molecules in the cell are seldom completely independent, network-based approaches have been adopted as the tools of choice to study such interconnected systems. Applied network-based methodologies have shown promising results in many branches of plant biology, including studies of immunity [21] and regulatory pathways [22,23]. When considering, for example, community enrichment [24], drug design [25] or the structural analysis of protein binding sites [26,27], network-based approaches have also been applied to organisms other than plants.

In this study, we adopted methods applied for the analysis of such information-rich structures to the modelling of BN-related natural events. The main contributions of this study are: (i) a methodology that considers temporal network community dynamics that is additionally enriched with domain background knowledge in the form of ontologies; (ii) a scalable algorithm for the reconstruction of scale-free networks that can be used to reconstruct networks from genome-wide RNA-Seq data; and (iii) the application of the developed modelling method to a dataset of grapevine samples infected with ‘*Ca*. P. solani’, through reconstruction of the networks.

## 2. Proposed Methodology

**Figure 1:**
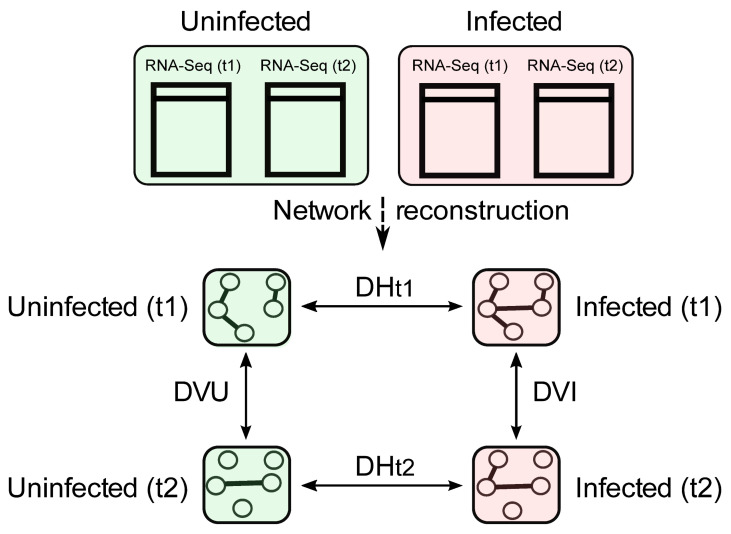
Schematic overview of the proposed approach. First, the RNA sequencing (RNA-Seq) expression networks are used to reconstruct four different regulatory networks, each corresponding to a particular phenotype uninfected with ‘*Ca*. P. solani’ (U) or infected with ‘*Ca*. P. solani’ (I), or the time of sequencing (t1, t2). The proposed methodology enables the exploration of eight main directions, depending on the time and phenotype considered (vertical lines, V), and also an exploration of differences between phenotypes within the same time frame (horizontal lines, H). D, dissipation, e.g., DVU, dissipation-vertical-uninfected; DVI, dissipation-vertical-infected.

An overview of the proposed methodology is shown in Figure 1 and is described in the following paragraphs.

### 2.1. Network Reconstruction

The purpose of network reconstruction is to explore higher-order feature (gene) interactions, attempting to overcome the independence assumption adopted many times in conventional statistical analysis. The field of network reconstruction has shown great promise for the analysis of biological data sets; namely, the methods such as LionessR [28], RAVEN [29,30], WGCNA and community-based reconstruction [31] were shown to accurately reconstruct yeast and human metabolic networks, offering novel insights into the space of potentially interesting biomarkers and new pathways.

The proposed methodology uses RNA-Seq expression data based on the normalised transcript counts, as commonly used throughout RNA-Seq experiments [32].

The expression data were initially pre-processed as follows. Only expressions, greater than a certain user-defined threshold, were used for edge construction—the process of inferring how a given pair of, e.g., genes (an edge), is connected. Here, 80% of the most expressed genes were selected, and the others were discarded (the 20th percentile was used as the threshold). This threshold was determined based on space requirements of the following steps in the network construction. For each gene, logarithms of the Euclidean distances betweenits and the remaining expression vectors were computed in a pairwise manner. The additional log step was performed due to high variability in the expression vectors, resulting in a large spectrum of possible distances at different scales. Hence, for each pair of genes, the distance that indicates the difference in their expression was recorded.

**Figure 2:**
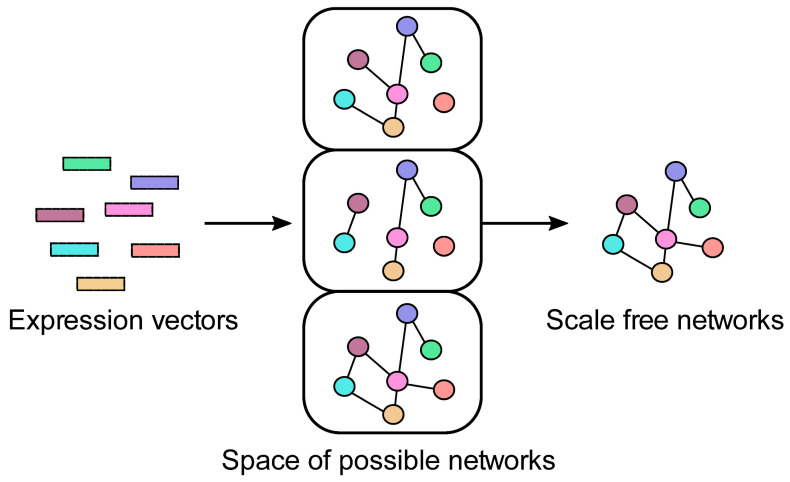
Schematic overview of the network (re)construction process.

The obtained matrix was then used for the reconstruction of the regulatory network (Figure 2). The key step in the network reconstruction is the identification of a distance threshold (i.e., the edge weight threshold), so that the resulting network is scale-free. By incrementally relaxing this threshold, more distant nodes become included in the final network, as lower distance thresholds permit the presence of edges corresponding to similar expression pairs. A search was implemented for possible networks, based on the previous studies of Rice et al. (2005) and Angulo et al. (2017) [33,34], with a comprehensive overview of the limitations of such approaches described elsewhere [35]. A user-specified interval of thresholds was automatically explored, where a network was constructed for each of the thresholds and was statistically evaluated for the scale-free properties. The scale-free networks are governed by the power-law distribution of the, e.g., node degrees in our case. This means that not all nodes are equally connected; thus, some are significantly more central than the other ones. Furthermore, in real-life scale-free networks, the presence of communities is often noted, because communities represent key functional aspects of a given network. The range of thresholds initially explored spanned from the 50th to the 99th percentiles of possible distances; however, this initial threshold range did not yield any scale-free networks and it was constrained to the final range between the 10th and 20th percentiles, which resulted in networks with distinct community structures suitable for further analysis. Although lower thresholds could be explored to include more less-expressed genes, the considered thresholds were within the limits of the used hardware, as described as follows. The processor used was an Intel(R) Xeon(R) Gold 6150 CPU @ 2.70 GHz (64 bit). Furthermore, the machine had 32 GB of RAM. Note that the worst-case computational complexity of the network construction is O(|N|^2), where N is the set of network nodes. Such graphs (cliques), albeit not being present in nature, can emerge as artefacts if too low thresholds (too many noisy genes) are considered, which we avoided with the considered threshold sets.

As soon as suitable scale-free properties were identified, the network search was terminated, and this constructed network was used for further analysis. The considered network reconstruction procedure was suitable for the following reasons. First, the initial attempts with correlation-based reconstruction yielded over-saturated networks, where too many links were created. Such networks had a negative impact on the subsequent community detection step’s computation time. Second, the scale-free test offered an automated procedure, which is more optimal than manual threshold identification, as more networks are explored. The scale-free assumption, however stringent, has been considered in the previous work [33,34] and yielded satisfactory networks. The distances were effectively computed via the SciPy package, version 1.5.4 and Numpy package, version 1.19.4. The storage of the networks was implemented via the NetworkX library, version 2.5 [36].

### 2.2. Community Enrichment

Conceptually, community-based semantic subgroup discovery (CBSSD) consists of two main steps, i.e., community detection followed by a one-versus-all enrichment procedure, which here, was additionally corrected for multiple hypothesis testing. We refer the reader to Škrlj et al. (2019) [24] for a more detailed overview of the enrichment process and for additional theoretical insights. Here, we used the method ‘as-is’, with the default hyperparameter settings including Bonferroni’s multiple test correction and the significance threshold of 0.05 (Fisher’s exact test). The tests were implemented via the statsmodels library, version 0.12.1 [37]. The background knowledge, however, needed to be specifically adapted, as grapevine is not a standard model organism, and as such, it is not well represented in conventional ontologies, such as Gene Ontology. For this purpose, we adopted the GoMapMan ontology, as discussed in Ramšak et al. (2014) [38].

#### 2.2.1. Community Detection

In the first step, the state-of-the-art community detection algorithm Infomap 1.3.1 [39] was used, resulting in nonoverlapping network partitioning—the clustering of the network nodes into units of hundreds of nodes (or more). The algorithm was run for 500 iterations with default hyperparameters to obtain stable community estimates. We compiled the C++ version of the stable release of the codebase found at https://github.com/mapequation/infomap (14 March 2021). The main rationale for this step was that communities were shown to correspond to functional network clusters; hence, performing the analysis at this level has the potential to detect sets of genes that are strongly associated with a given process. Once the communities were identified, we proceeded to work with those comprising more than five nodes but no more than 30. The rationale behind this decision was to emphasise communities with a significant functional annotation that could also be inspected by a domain expert, as very large communities are not necessarily easily inspectable and associated with specific processes. However, finding communities with still unknown associations with specific processes was a key focus of this study.

#### 2.2.2. Community Enrichment

**Figure 3:**
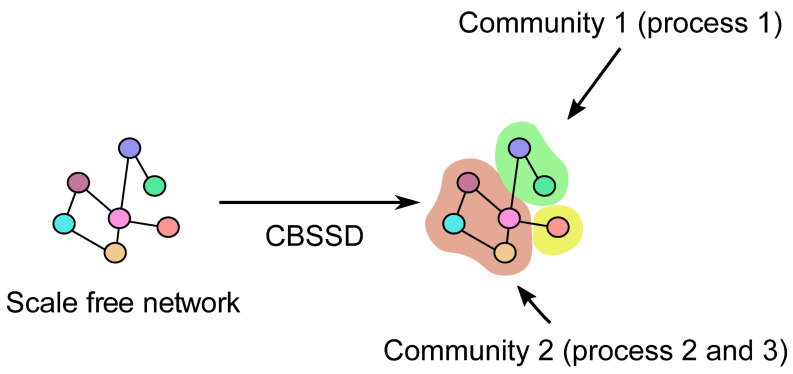
Overview of the community-based semantic subgroup discovery (CBSSD) methodology. Given a complex network, CBSSD offers the functional enrichment of communities within the network. The functional enrichment corresponds to the assignment of expert-derived process descriptions to parts of the communities.

Communities were subjected to standard term enrichment, where Fisher’s exact tests were used to determine whether a given semantic term (i.e., specific process in Figure 3) can be associated with particular communities, and not to others. The *p*-values obtained from the Fisher’s exact tests were additionally corrected using Bonferroni’s multiple test corrections, to allow for simultaneous testing of multiple hypotheses.

### 2.3. Community Dissipation

**Figure 4:**
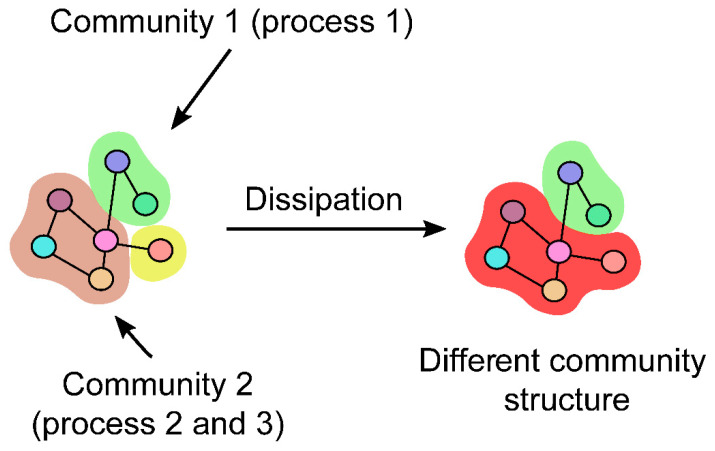
Community dissipation. Community dissipation corresponds to the measurement of how different a given community is across a pair of time points. For example, here, the brown (**left**) community becomes part of the red community (**right**), where the yellow community with a single node (**left**) disappears and becomes part of the red community (**right**).

One of the novel contributions of this work is the concept of community dissipation (Figure 4), as the process of the loss of integrity of a given community. In this section, we first present the formal definition of the dissipation index, followed by its generalisation to arbitrary time series of (multiplex) networks. We next present an algorithm that computes the proposed measure efficiently.

#### Dissipation between Two Time Points

Let G1 = (N, E1) and G2 = (N, E2) represent two undirected networks that correspond to two time points, t1 and t2. Let φ: G → P represent the mapping from a network to a given partition of the network nodes. We refer to this mapping as community detection. Assume *P*1 and *P*2 represent two partitions, N1 and N2, respectively. We are interested in the elements of *p* ∈ *P*1, when observed together in *P*2. We propose a measure that summarises the behaviour of all of the communities (*P*1) when their elements are assessed in *P*2. Intuitively, the proposed community dissipation operates as follows. For each node of a given community, *p*, its presence in the second network communities is recorded. For each community of the second network that contains at least one node from the origin community, the fraction of the covered community is recorded. We refer to this score as the coverage (*cov*), defined as in Equation (1):

(1) $$cov\left(p,P2\right)={\left\{\frac{\left|k\cap p\right|}{\left|p\right|};k\cap p\neq \emptyset \right\}}_{k\in P2}$$

We can finally compute the dissipation index (*dis*), as in Equation (2):

(2) $$dis\left(p,P2\right)=-ln\left(\frac{max\left[cov\left(p,P2\right)\right]}{\left|cov\left(p,P2\right)\right|}\right)$$

This index thus results in high values if the observed community is different in the consequent time point—either it is smaller or it is absorbed into a larger community. Both events result in high dissipation. Similarly, low dissipation indicates a stable community structure. The dissipation index is used alongside the enrichment analysis to assess temporal changes at the functional level. The proposed dissipation index is complementary to the manual inspection of communities at different time points that can be a cost- and time-consuming process. An alternative approach to using the dissipation index can include temporal community detection [40]; however, this method is not necessarily suitable for the data-scarce regime with only two time points considered in this work. The indication that the post-hoc analysis of communities is more suitable for analysis was also recently demonstrated when studying fire events in a portion of the Amazon basin [41].

### 2.4. Applications of the Methodology

There are several distinct possible applications of this methodology. An outline of one possible use is shown in Figure 1, and it is discussed in more detail in this section.

#### 2.4.1. Temporal Enrichment

Given a pair of networks, the proposed methodology allows the exploration of communities within these networks that persist (i.e., have low dissipation indices) and those that break apart (i.e., have high dissipation indices). For example, the first network corresponds to the set of grapevine samples collected early in the growing season, and the second network corresponds to the set of grapevine samples collected late in the growing season. As the raw information obtained was not directly useful for domain experts, the CBSSD step used for the enrichment of the first network offers additional functional insight that would otherwise not be accessible. For example, the monitoring of a community that is distinctly described using terms related to the process of photosynthesis or carbohydrate metabolism can be assessed.

#### 2.4.2. Phenotype Comparison

An alternative application of the proposed methodology relates to a pair of different phenotypes at the same time, e.g., uninfected grapevine samples and grapevine samples infected with ‘*Ca.* P. solani’. In this case, enrichment via CBSSD is conducted in the same manner as for the temporal enrichment; however, the communities are compared with respect to a given phenotype and not to time. Such comparisons can unveil communities that are coherent in uninfected plants, but dissipated in infected plants, or vice versa. Note that two such comparisons need to be manually inspected by the domain experts. The code to replicate the methodology is freely available at: https://gitlab.com/skblaz/community-enrichment-phytoplasma (accessed on 20 March 2021).

## 3. Evaluation of the Methodology

The proposed methodology was applied to a subset of the RNA-Seq data set of uninfected and infected grapevine (*Vitis vinifera*) cv. Zweigelt samples collected from a production vineyard. In the present study, we focused on the samples collected late in the growing season, when symptoms of BN were prominent on the grapevines infected with ‘*Ca*. P. solani’. The sample descriptions and processing are described in detail in the Supplementary Methods. Gene set enrichment analyses, multidimensional scaling and principal component analysis based on the same samples were performed in the frame of study by Dermastia et al. 2021 [42]. In this study, normalised counts were subjected to the proposed methodology described in Section 2.

This analysis demonstrated not only some associations that were known from previous studies of BN, but also some new associations, which can serve for the design of novel studies (Table 1).

### 3.1. Recovery of Empirically Validated Community Information

**Figure 5:**
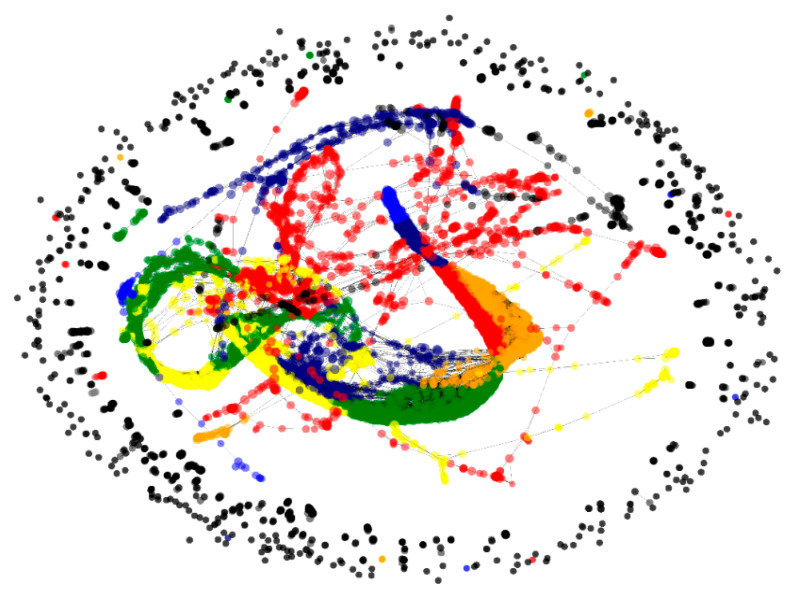
Reconstructed network where several communities of genes from different metabolic pathways are formed. These were visualised with Py3plex and are shown in different colours.

After the application of a new modelling method to the transcriptional data of these samples, several communities of genes from different metabolic pathways were formed and visualised with Py3plex [43] (Figure 5).

There is growing evidence that the phytoplasma infection of grapevines is characterised by severely affected photosynthesis and carbohydrate metabolism pathways [16,38]. In good correlation with these data, the present modelling of the data from the samples infected with ‘*Ca.* P. solani’ late in the growing season revealed two main communities that were associated with these two pathways. At the late growing season time point, these communities significantly disintegrated with a dissipation index of 0.48 (highest 30% of all dissipations) for the uninfected samples (Table 1). The communities revealed comprised some genes associated with photosynthesis or carbohydrate metabolism that have been detected before in phytoplasma-infected plants. Their re-discovery by the applied model assured us that the method is relevant for such analysis, and that new genes that have not yet been associated with these processes and were involved in these communities might also have biologically meaningful functions. Moreover, they may present a new reference framework for further research of phytoplasma-infected plants.

#### 3.1.1. A Community of Genes Associated with Photosystem II

In previous studies of phytoplasma diseases in general, and of BN in particular, it has been shown that several genes or their protein products involved in different steps of photosynthesis were down-regulated upon phytoplasma infection [10,12,44,45,46]. Among these, the transcript levels of 11 genes that encode some of the most abundant proteins of the chloroplasts, chlorophylls a/b binding proteins in photosystem II, were significantly decreased in the leaves of grapevine cv. Chardonnay infected with ‘*Ca.* P. solani’ [10]. Some of the genes that encode chlorophyll a/b binding proteins were also down-regulated in phytoplasma-infected coconut, and the chlorophyll a/b binding protein 5 protein levels were lower in phytoplasma-infected *Paulownia fortunei* [47,48]. Consistent with previous results is the detected significant down-regulation of *Vitvi10g00740*, which encodes chlorophyll a/b binding protein 1, in grapevine cv. Zweigelt infected with ‘*Ca.* P. solani’ in comparison with the uninfected grapevines (Table 1).

Another gene detected in the community was *Vitvi11g00097*, which encodes a MYB domain transcription factor, as one of several regulators of the general branch and different branches of flavonoid biosynthesis. Its transcript levels significantly increased in the grapevines infected with ‘*Ca.* P. solani’, compared to the uninfected grapevines (Table 1). It can be noted that several genes involved in flavonoid biosynthesis (as well as their products) have been shown to be up-regulated in grapevines infected with ‘*Ca.* P. solani’ [9,16]. While *Vitvi11g00097* has never been associated with phytoplasma infections of grapevine before, it shows large divergent changes in its transcript levels during grapevine berry development and under different light-exposure treatments of the grapevines [49]. Its high increase in expression suggests an important role in phytoplasma pathogenicity. A significant up-regulation was also detected for a gene from the cytochrome P450 superfamily (Table 1). This is an ancient superfamily that has been identified in all domains of organisms [50]. Its members are involved in multiple metabolic pathways with distinct and complex functions, and they have important roles in a vast array of reactions. As a result, in plants, numerous secondary metabolites are synthesised that function as growth and developmental signals, or that can protect plants from various biotic and abiotic stresses [50]. The expression of the cytochrome P450 genes shows temporal variation [51]. In association with phytoplasmas, they have been shown to have a role in the ‘*Ca. P. ulmi*’-infected leafhopper *Amplicephalus curtulus* [52].

The ubiquitous enzyme dihydrofolate reductase is a key enzyme in the folate biosynthetic pathway [53], and it has an essential role in the synthesis of DNA precursors and some amino acids [54]. Despite its importance, information about plant dihydrofolate reductase is scarce [53]. On the other hand, it is known that humans and other animals cannot synthesise folates *de novo*, and thus rely on their diets for folate intake [55]. Moreover, the completely sequenced phytoplasma genomes [56] provide evidence that phytoplasmas are experiencing an ongoing evolutionary process, whereby they are losing the ability to synthesise folate, and consequently, they must rely on their host for folate repletion [57]. How the increase in dihydrofolate reductase gene transcript levels in infected grapevines compared to uninfected grapevines (Table 1) are related to the requirement of phytoplasma to obtain folate is an interesting point for further research. In addition, the role of the significantly up-regulated gene *Vitvi05g01860* (Table 1) is currently unknown.

#### 3.1.2. A Community of Genes Associated with Pathways That Are Usually Not Considered to Interact Directly

**Figure 6:**
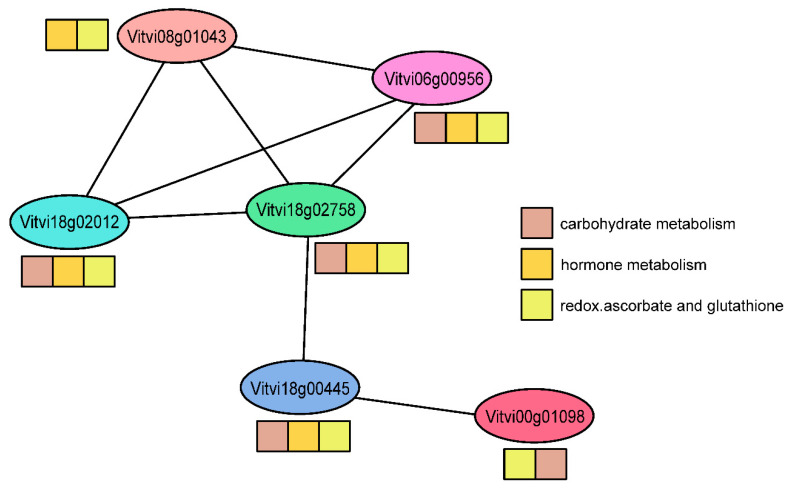
A community of six genes associated with three different metabolic processes correspond to Community 2 in Table 1. The coloured squares indicate gene-process associations.

The second community detected by this modelling is more complex (Table 1, Figure 6), and comprises several genes that are associated with different metabolic pathways, including starch biosynthesis, abscisic acid synthesis/degradation, ascorbate, and glutathione. As a proof of concept, in this community, the modelling revealed the up-regulation of the gene that encodes a large subunit of ADP-glucose pyrophosphorylase (AGPase) (Table 1). This transcript is involved in starch biosynthesis, and its increase has been shown before in grapevines infected with phytoplasmas. The increased expression of this AGPase gene was detected in grapevine cv. Chardonnay infected with ‘*Ca*. P. solani’ [20], as well as in cv. ‘Modra frankinja’ (syn. ‘Blaufränkisch’) infected with phytoplasma Flavescence dorée, where the activity of its corresponding enzyme was also detected, together with increased starch concentrations [58]. Similarly, a role for ascorbate peroxidase (Table 1) in grapevine phytoplasma infections has been shown before [59,60,61,62].

Additional genes revealed by the applied method in the second community have not been considered in plants infected with phytoplasmas nor in metabolic processes that interact directly. A significantly up-regulated gene in this community encodes for a protein from the RING/U-box superfamily (Table 1). In *Arabidopsis thaliana*, this gene was reported to be an effector of abscisic acid accumulation after induced drought [63]. Therefore, the association of the RING/U-box superfamily protein with the abscisic acid pathway in grapevines infected with ‘*Ca.* P. solani’ seen here is of importance. Previously, there were only two studies of phytoplasma-infected paulownia plants that identified some differentially expressed genes or proteins involved in abscisic acid metabolism, which were based on high-throughput RNA-Seq and proteomic analysis, although these provided no verification of their clear function [64,65].

In all living organisms, many cellular signal transduction pathways are mediated by receptor-like kinases. The largest group of plant receptor-like kinases is the leucine-rich repeat receptor-like kinases [66], which have roles in plant development and the defence against pathogens [67,68,69]. The leucine-rich repeat receptor-like kinase family protein was up-regulated in the infected grapevine samples in the present study (Table 1). However, the detection in this community and the biological function here are currently not clear.

#### 3.1.3. Community Enrichment and the Classical Differential Expression Analysis

The proposed methodology operates at the level of individual community-term associations. To further study the relation between the conventional enrichment and the enriched communities, we performed additional analyses discussed next.

First, we compared the community-based results to those obtained by a differential expression analysis (detailed in Supplemental Table S1). To see whether our community approach captures a higher-than-expected number of differentially expressed genes, a 2 × 2 contingency table was prepared to compare differentially and nondifferentially expressed genes present in the gene list that we obtain from a differential expression analysis (Supplemental Table S1A) and genes present in our calculated communities (Supplemental Table S1B,C). Prior to that, genes from communities of both directions (Table S1B, uninfected vs. infected; Table S1C; infected vs. infected) were merged into a single list (as differential expression analysis detects differentially expressed genes in both directions as well; Table S1A) and analysed as a whole. Fisher’s Exact Test for Count Data was used, where we observed that there are significantly more differentially expressed genes present in the final set of communities when compared to the remaining set of genes from a differential expression analysis (corrected *p*-value of 2.084 × 10^−14^). This result indicates that differentially expressed genes are more likely to be present in multi-gene communities than not, where they potentially act as key nodes that maintain a given community’s structure. Although communities cover a smaller amount of genes (310; Table S1B,C) compared to the full set of differentially expressed genes (FDR *p*-value ≤ 0.05; 6115 differentially expressed genes; Table S1A), it represents a complementary method from the interpretation standpoint, similarly to the, e.g., gene set enrichment analysis.

**Figure 7:**
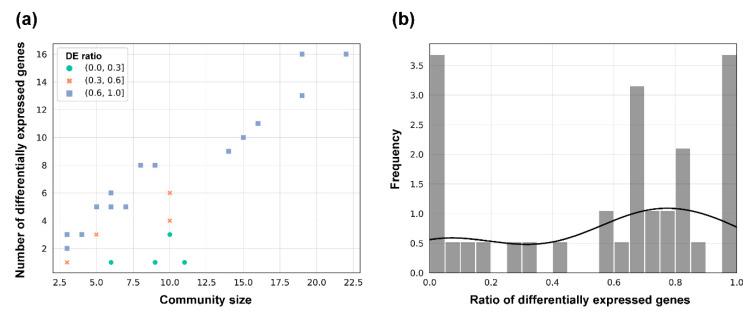
(**a**) Relationship between the community size and number of differentially expressed (DE) genes in that community (Supplemental Table S1B,C). (**b**) Histogram of the distribution of the proportions of differentially expressed genes (number of bins: 20. The ‘Frequency’ corresponds to the density estimated via the seaborn package).

In the second step, we aimed to observe the proportion of differentially expressed and differentially nonexpressed genes in each community separately (Supplemental Table S1B,C). Results show that the method is not only able to capture communities with a high number of differentially expressed genes (14 out of 39, where >75% of genes in the community are differentially expressed), but it also captures many (7 out of 39) that do not contain a single differentially expressed gene (Figure 7a). In addition, while the method does result in communities of varying sizes, the proportion of differentially expressed genes does rise with respect to the detected community size.

The results of the additional statistical analysis presented in this section indicate that the community structure indeed entails many significantly expressed genes, and as such, offers an additional layer of information that potentially facilitates the final interpretation of the gene roles and facilitates the search of novel hypotheses (possible interactions). As such, it has the potential to notably reduce the amount of manual work (and hence time) of a domain expert to identify potentially interesting relations between genes, and as such, represents a viable complementary method to conventional analysis.

## 4. Validation of the Results

To experimentally validate the obtained results, we have chosen a glutathione-S-transferase gene. It was associated with a community that included genes related to photosystem II of photosynthesis and was strongly transcriptionally activated by ‘*Ca.* P. solani’ infection (Table 1). Increases in the transcript of a glutathione-S-transferase gene and its protein product have already been shown to occur during phytoplasma infection of the Chinese jujube, which results in jujube witches’ broom disease [70]. The plant glutathione S-transferases comprise a large family of ubiquitous multifunction proteins that contribute to the detoxification of endogenous or xenobiotic compounds and oxidative stress metabolism. They are induced under several stress conditions, including microbial infections [71]. However, their role associated with photosynthesis has not been shown before in phytoplasma-infected plants.

Therefore, the transcript increase in the glutathione S-transferase gene was additionally independently empirically validated by measurement of the enzymatic activity of the glutathione S-transferase protein (Supplementary Methods).

**Figure 8:**
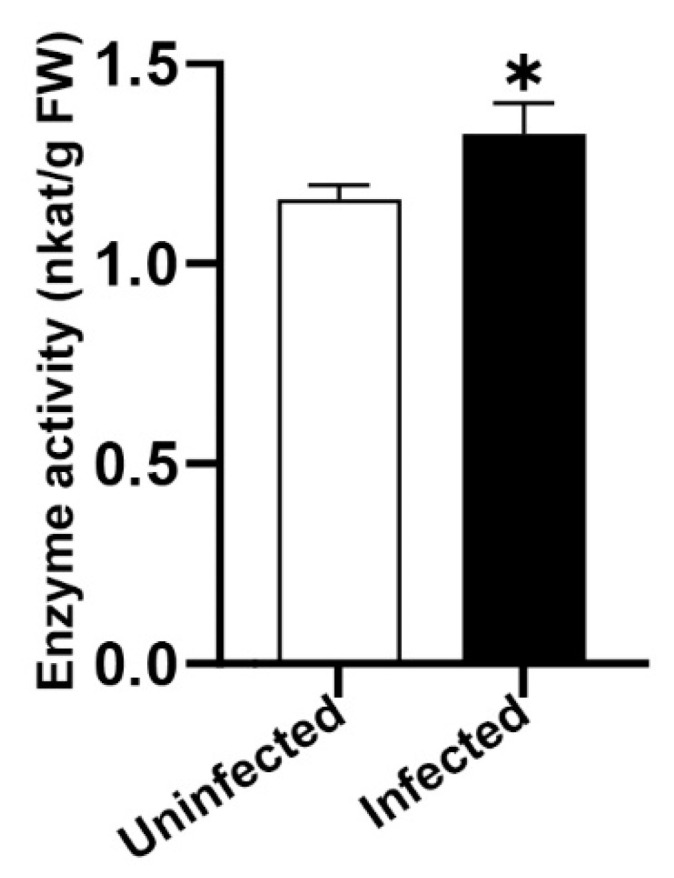
Empirical validation of the glutathione S-transferase activity in uninfected and infected with ‘*Ca*. P. solani’ grapevines cv. Zweigelt in the late growing season. FW, fresh weight; the data refer to means ± standard deviation. * *p* < 0.05 (Mann-Whitney test).

A higher enzyme activity was seen for these grapevines infected with ‘*Ca.* P. solani’ (Figure 8), which is in agreement with the transcriptional activation of the glutathione-S-transferase gene upon infection (Table 1). The combined results suggest an important role for glutathione-S-transferase in impaired photosynthesis shown before in grapevines infected with ‘*Ca.* P. solani’ [10,12,44,45,46]. This novel actor may open new routes for the research of phytoplasmas.

## 5. Discussion

Here, we have proposed a methodology for network enrichment based directly on RNA-Seq data. The methodology was used to model BN phytoplasma-infected grapevines to provide novel insights into the biochemical mechanisms that potentially differentiate healthy grapevines from infected grapevines. The proposed methodology offers both the confirmation of existing empirical data and potentially interesting novel candidates that appear to be associated with the processes studied. The methodology is one of the first direct approaches that join network reconstruction with network enrichment, and it provides the potential for end-to-end analysis at the network level, directly from high-throughput RNA-Seq data.

Although the method indeed offers empirically provable results, it still has its limitations. The initial step of network reconstruction is computationally feasible under the following assumptions. The Euclidean distances between the expression vectors should be representative for the modelling of real relations between the genes. Furthermore, the key assumption that offers the filtering of potentially interesting networks from those that appear to not be interesting is that the networks are scale-free. As the statistical test used to assess the scale-free properties is rapid, many candidate networks can be inspected. The resulting networks are at least approximately scale-free (if no real scale-free network is found); however, the phenomenon studied might not give rise to scale-free networks [72], in which case the proposed methodology will not retrieve the best candidates, although it will still offer feasible candidates.

The proposed methodology, albeit based on many necessary assumptions, offers insights into the interactions between the genes. As such, it transcends the independence assumption commonly adopted in classical analysis, and it offers insights into potentially more interesting regulatory mechanisms. The proposed work demonstrates that reconstruction-based enrichment can offer novel candidate genes that were also shown to behave accordingly when tested in vitro. When comparing the community-based analysis used in this work with the conventional gene set enrichment analysis that determines whether an a priori defined set of genes shows statistically significant, concordant differences between two biological states, we observed that many genes, present in the enriched communities, were in fact not individually enriched via the conventional analysis, where expression vectors are compared individually relative to the others. For this observation, there can be multiple explanations, including the following ones. First, given that the community enrichment considers only the counts of a particular annotation within/outside a given community, this step is highly dependent on the structure of the considered network. As the networks are derived via the threshold-based filtering procedure, for which it is known that small changes in the threshold can have a great impact on the network itself, the network generation process can have a substantial effect on the results of enrichment. This is the trade-off when comparing the proposed method to conventional enrichment, which is structure-independent, whilst simultaneously being unable to operate in the space of interactions. The second main reason for the observed discrepancy could be the impact of the type of test and the corresponding multiple test correction used. Although the type of *p*-value correction is a free parameter of the method, we believe that more involved correction schemes beyond the Bonferroni correction could be explored to further assess the consistency of the results. We are by no means claiming that the hyperparameter setting, which yielded promising results in this study, generalises to unknown reconstruction/enrichment problems; individual expression-based problems are most probably subject to different underlying regulatory structures, which should be explored on a per-problem basis.

Note, however, that although the proposed methodology is capable of operating with a potentially interesting space of e.g., gene–gene interactions, this does not guarantee the causality of such interactions. As long as the distance-based reconstruction of the regulatory networks is considered without additional constraints based on, e.g., existing empirical evidence (measured interactions), the methodology effectively serves as a hypothesis generation engine, offering insight into structures that emerge from the reconstructed regulatory networks, albeit some of them being artefacts of the method. For example, the community detection method employed can have different sensitivities (resolution limits) when considering smaller communities. We attempted to overcome this issue by selecting a method which is known to perform well in such settings; however, the considered method (InfoMap) could be unsuitable for a related domain where a different type of community detection could be more useful.

Finally, we consider the developed methodology as complementary to many other established approaches based on frequentist statistics, as the network-based approach can unveil novel hypotheses that are not reachable via, e.g., individual-level enrichment analysis. Further work also includes extending the network generation process with measures of network segmentation such as the clustering coefficient, which could further improve the quality of the reconstructed networks.

## Figures and Tables

**Table 1:** Examples of the enriched communities with a direction of analysis from infected to uninfected grapevines late in the growing season, where the dissipation index was 0.48. Each community was annotated with the members from the GoMapMan ontology [38], providing direct insight into their associated processes. The analysed data were differentially expressed genes from uninfected grapevines cv. Zweigelt and from samples infected with ‘*Ca.* P. solani’. For each mRNA sequence, the differences in expression between phytoplasma-infected and -uninfected plants were calculated as log_2_FC. Only mRNAs with a false discovery rate (FDR) adjusted *p*-value < 0.05 were considered as differentially expressed. U, uninfected samples; I, samples infected with ‘*Ca*. P. solani’.

Community	Bin Annotating the Community	Gene ID	RNA Description	log_2_ FC I: U	Adjusted *p*-Value (FDR)
1	1.1.1.1 PS.lightreaction.photosystem II.LHC-II	*Vitvi07g03081*	Cinnamyl alcohol dehydrogenase 8|Chr4:17855964-17857388 FORWARD LENGTH = 359|201606	−1.40	0.058
	1.1.1.1 PS.lightreaction.photosystem II.LHC-II	*Vitvi01g01482*	BHLH transcription factor-like protein	−2.15	0.003
	1.1.1.1 PS.lightreaction.photosystem II.LHC-II	*Vitvi08g02107*	Dihydrofolate reductase|Chr4:12612554-12613586 FORWARD LENGTH = 261|201606	2.31	0.000
	1.1.1.1 PS.lightreaction.photosystem II.LHC-II	*Vitvi07g02188*	Glutathione S-transferase family protein|Chr3:23217425-23218246 REVERSE LENGTH = 219|201606	3.24	0.000
	1.1.1.1 PS.lightreaction.photosystem II.LHC-II	*Vitvi10g00740*	Chlorophyll A/B binding protein 1|Chr1:10478071-10478874 FORWARD LENGTH = 267|201606	−2.80	0.001
	1.1.1.1 PS.lightreaction.photosystem II.LHC-II	*Vitvi11g00097*	Unknown protein	4.36	0.000
	1.1.1.1 PS.lightreaction.photosystem II.LHC-II	*Vitvi03g01524*	Cytochrome P450%2C family 82%2C subfamily C%2C polypeptide 2	2.73	0.000
	1.1.1.1 PS.lightreaction.photosystem II.LHC-II	*Vitvi05g01860*	No description	4.55	0.026
	1.1.1.1 PS.lightreaction.photosystem II.LHC-II	*Vitvi16g00810*	Protein kinase superfamily protein|Chr1:24961634-24963941 REVERSE LENGTH = 663|201606	−3.20	0.000
2	2.1.2.1 major CHO.metabolism.synthesis.starch.AGPase	*Vitvi00g01098*	Leucine-rich receptor-like protein kinase family protein|201606	1.30	0.002
	2.1.2.1 major CHO.metabolism.synthesis.starch.AGPase	*Vitvi18g02758*	ADPGLC-PPase large subunit	1.25	0.002
	2.1.2.1 major CHO.metabolism.synthesis.starch.AGPase	*Vitvi06g00956*	Aldehyde oxidase 1	−0.86	0.036
	2.1.2.1 major CHO.metabolism.synthesis.starch.AGPase	*Vitvi18g00445*	Ascorbate peroxidase 4|Chr4:5777502-5779064 REVERSE LENGTH = 284|201606	−1.64	0.000
	2.1.2.1 major CHO.metabolism.synthesis.starch.AGPase	*Vitvi18g02012*	UDP-glucosyl transferase 88A1|Chr3:5618847-5620833 REVERSE LENGTH = 446|201606	−1.31	0.005
	17.1.1.1.12 hormone metabolism.abscisic acid.aldehyde.oxidase	*Vitvi08g01043*	RING/U-box superfamily protein|Chr5:24354298-24356706 FORWARD LENGTH = 487|201606	1.79	0.000
	17.1.1.1.12 hormone metabolism.abscisic acid.aldehyde.oxidase	*Vitvi18g02758*	ADPGLC-PPase large subunit|Chr1:9631630-9634450 FORWARD LENGTH = 518|201606	1.25	0.002
	17.1.1.1.12 hormone metabolism.abscisic acid.aldehyde.oxidase	*Vitvi06g00956*	Aldehyde oxidase 1|Chr5:7116783-7122338 FORWARD LENGTH = 1368|201606	−0.86	0.036
	17.1.1.1.12 hormone metabolism.abscisic acid.aldehyde.oxidase	*Vitvi18g00445*	Ascorbate peroxidase 4|Chr4:5777502-5779064 REVERSE LENGTH = 284|201606	−1.64	0.000
	17.1.1.1.12 hormone metabolism.abscisic acid.aldehyde.oxidase	*Vitvi18g02012*	UDP-glucosyl transferase 88A1|Chr3:5618847-5620833 REVERSE LENGTH = 446|201606	−1.31	0.005
	21.2.1 redox.ascorbate and glutathione.ascorbate	*Vitvi00g01098*	Leucine-rich receptor-like protein kinase family protein|201606	1.30	0.002
	21.2.1 redox.ascorbate and glutathione.ascorbate	*Vitvi08g01043*	RING/U-box superfamily protein|Chr5:24354298-24356706 FORWARD LENGTH = 487|201606	1.79	0.000
	21.2.1 redox.ascorbate and glutathione.ascorbate	*Vitvi18g02758*	ADPGLC-PPase large subunit|Chr1:9631630-9634450 FORWARD LENGTH = 518|201606	1.25	0.002
	21.2.1 redox.ascorbate and glutathione.ascorbate	*Vitvi06g00956*	Aldehyde oxidase 1|Chr5:7116783-7122338 FORWARD LENGTH = 1368|201606	−8.86	0.036
	21.2.1 redox.ascorbate and glutathione.ascorbate	*Vitvi18g00445*	Ascorbate peroxidase 4|Chr4:5777502-5779064 REVERSE LENGTH = 284|201606	−1.64	0.000
	21.2.1 redox.ascorbate and glutathione.ascorbate	*Vitvi18g02012*	UDP-glucosyl transferase 88A1|Chr3:5618847-5620833 REVERSE LENGTH = 446|201606	−1.31	0.005

## Data Availability

The source data for this study have been deposited in the European Nucleotide Archive (ENA) in the frame of another study [Dermastia et al., 2021 (submitted)] in fastq format under project accession PRJEB42777 and samples accessions: ERS5673304, ERS5673305, ERS5673306, ERS5673307, ERS5673308, ERS5673309, ERS5673310, and ERS5673311. Code to replicate the methodology is freely available at: https://gitlab.com/skblaz/community-enrichment-phytoplasma (accessed on 20 March 2021).

## Supplementary Materials

Supplementary materials can be found at https://www.mdpi.com/article/10.3390/plants10040646/s1. Supplementary Methods. Supplementary Table S1.
