# Supplementary material for: New Cross-Talks between Pathways Involved in Grapevine Infection with ‘Candidatus Phytoplasma solani’ Revealed by Temporal Network Modelling
Source: Plants (Basel). 2021 Mar 29;10(4):646. doi: 10.3390/plants10040646 (PMC8065506; doi:10.3390/plants10040646)
Supplement: Supplementary file 1 [file plants-10-00646-s001.zip › plants-1114509_RESUBMISSION2_Supplementary Methods.pdf]

Supplementary Methods

Plant material

Four phytoplasma-infected grapevines (*Vitis vinifera*) cv. Zweigelt (Rotburger) and their four healthy neighbours from a vineyard in Klosterneuburg (Austria) were selected for the study, according to the development of their symptoms in the previous season (2016). The grapevines were planted in 2006, and the rootstock is Kober 5 BB. The first fully developed leaves were sampled from several canes of each vine in June and September 2017. The leaf veins were cut out of the leaves sampled, flash-frozen in liquid nitrogen, ground to a fine powder, and stored at -80 °C.

Detection of phytoplasma

In September 2017, all four phytoplasma-infected grapevines showed typical symptoms of infection including reddening and curling of leaves and no or incomplete fruit development. The presence of '*Ca*. P. solani' was confirmed by PCR with *SecY*, *Stamp*, *tuf* and *vmp1* specific primers as described previously [1]. Based on these four markers the previously described nettle type CPsM4_At1 was detected for all four of the infected grapevines. For the four control grapevines, none of the markers gave any PCR signal. All of the grapevines were free of symptoms of powdery and downy mildew. ESCA symptoms had not been recorded for any of the tested grapevines since 2006.

RNA extraction and sequencing

Total RNAs were extracted from sampled leaf veins, using an optimised cetyltrimethylammonium bromide (CTAB) method (adapted from [2]) combined with RNA purification on columns (Zymo-Spin; Direct-zol RNA MiniPrep Plus kits, Zymo Research). About 50 mg frozen and powdered tissue was further homogenised with steel beads for 10 min at maximum speed in 800 µL CTAB buffer (100 mM Tris-HCl, pH = 8, 2 M NaCl, 25 mM EDTA, 2.0% [w/v] CTAB, 2.5% [w/v] PVP40, 2.0% [v/v] β-mercaptoethanol) using TissueLyser (Qiagen). After the addition of an equal volume of chloroform-isoamyl alcohol (24:1), the sample was vortexed and centrifuged for 10 min at 10,000× g at 4 °C. The upper aqueous phase was recovered, to which 1.5 volume of pure ethanol was added. After a 30 min precipitation at 4 °C, the mixture was transferred into the columns (Zymo-Spin). The RNA was purified according to the manufacturer instructions, with an additional washing step and a second prewashing step added to the beginning of the purification process. To elute the RNA, 30 µL preheated (80 °C) DNase/RNase-free water was added to the column and incubated for 10 min at room temperature. This was followed by 1 min centrifugation at 14,000× g. The isolated RNA was subjected to DNase digestion (DNase I Set; Zymo Research), and cleaned up using RNA Clean & Concentrator kits (Zymo Research). RNA concentration, integrity and purity were assessed using a Bioanalyser (2100) and RNA 6000 Nano kits (Agilent Technologies). Library preparations for the mRNAs sequencing services (Illumina HiSeq 4000), and preprocessing to remove the adapter sequences and low-quality reads were provided by Novogene (Hong Kong).

mRNA data analysis

The 150 bp paired-end reads obtained were trimmed to remove low-quality bases (Phred <20), clipped to remove the remaining adapter sequences, and mapped to the 12X.2 version of the PN40024 grapevine reference genome (https://urgi.versailles.inra.fr/files/Vini/Vitis%2012X.2%20annotations/), using CLC Genomics Workbench 12.0 (Qiagen), with the following parameters: mismatch cost, 2; insertion or deletion cost, 3; length fraction, 1.0; similarity fraction, 0.95; and maximum number of hits for a read, 1. The reads were annotated using the VCost.v2 annotation. The raw counts from the transcripts were exported and have been deposited with the European Nucleotide Archive, under project accession number PRJEB42777.

The differential expression analysis was performed in R v3.4.2 [3], using the *limma* package v3.34.9 [4].

mRNA counts with a baseline expression level of >50 reads mapped in at least 4 samples were TMM-normalised in edgeR v3.20.9 [5] and transformed using *voom* [6]. To identify differentially expressed mRNAs, the empirical Bayes approach was used, with Benjamini and Hochberg's (FDR) p-value adjustment. Genes with adjusted p-values <0.05 were considered statistically significantly differentially expressed.

Measurement of the glutathione S-transferase activity

Glutathione S-transferase was extracted from fresh frozen uninfected and with '*Ca*. P. solani' infected leaf grapevine samples of cv. Zweigelt collected late in the growing season exactly as previously described [7], and adjusted according to Anžič [8]. The enzymatic activity assays were performed in UV-transmissive, flat-bottomed, 96-well plates (UV-Star Greiner Bio One; Kremsmünster, Austria). Protein extract volumes from 1 μL to 20 μL were used for the reactions. The total reaction volume was 160 μL. Reaction mixes were incubated in a plate reader (Ascent Multiskan; Thermo Fisher Scientific) for 40 min at 25 °C or 30 °C, according to the optimised protocol for each enzyme. All of the assays were carried out in triplicate, and for the control assays the substrate was not added to the reaction mixes. Changes in absorbance per second were used to calculate the activities of the enzymes, as nkat/g fresh weight. Glutathione S-transferase activity was measured in samples incubated with 1 mM 2,4-dinitrochlorobenzene and 1 mM reduced glutathione in 100 mM K2HPO4/KH2PO4 buffer, pH 7.4. 2,4-Dinitrochlorobenzene was omitted for the control reactions. Absorbance was measured at 334 nm, with the formation of (2,4-dinitrophenyl) glutathione. The enzymatic activities were further corrected by subtracting the non-enzymatic formation of (2,4-dinitrophenyl) glutathione by including in the 96-well plate a column without any extract added.

References

1. Aryan, A.; Brader, G.; Mörtel, J.; Pastar, M.; Riedle-Bauer, M. An abundant '*Candidatus* Phytoplasma solani' tuf b strain is associated with grapevine, stinging nettle and Hyalesthes obsoletus. *Eur. J. plant Pathol. / Eur. Found. Plant Pathol.* **2014**, *140*, 213–227, doi:10.1007/s10658-014-0455-0.

2. Carra, A.; Gambino, G.; Schubert, A. A cetyltrimethylammonium bromide-based method to extract low-molecular-weight RNA from polysaccharide-rich plant tissues. *Anal. Biochem.* **2007**, *360*, 318–320, doi:10.1016/j.ab.2006.09.022.

3. R Core Team (2017) R A Language and Environment for Statistical Computing. - References - Scientific Research Publishing Available online: https://www.scirp.org/(S(351jmbntvnsjt1aadkposzje))/reference/ReferencesPapers.aspx?ReferenceID=2144573 (accessed on Jan 21, 2021).

4. Ritchie, M.E.; Phipson, B.; Wu, D.; Hu, Y.; Law, C.W.; Shi, W.; Smyth, G.K. Limma powers differential expression analyses for RNA-sequencing and microarray studies. *Nucleic Acids Res.* **2015**, *43*, e47, doi:10.1093/nar/gkv007.

5. Robinson, M.D.; McCarthy, D.J.; Smyth, G.K. edgeR: A Bioconductor package for differential expression analysis of digital gene expression data. *Bioinformatics* **2009**, *26*, 139–140, doi:10.1093/bioinformatics/btp616.

6. Law, C.W.; Chen, Y.; Shi, W.; Smyth, G.K. Voom: Precision weights unlock linear model analysis tools for RNA-seq read counts. *Genome Biol.* **2014**, *15*, R29, doi:10.1186/gb-2014-15-2-r29.

7. Jammer, A.; Gasperl, A.; Luschin-Ebengreuth, N.; Heyneke, E.; Chu, H.; Cantero-Navarro, E.;

Großkinsky, D.K.; Albacete, A.A.; Stabentheiner, E.; Franzaring, J.; et al. Simple and robust determination of the activity signature of key carbohydrate metabolism enzymes for physiological phenotyping in model and crop plants. *J. Exp. Bot.* **2015**, *66*, 5531–42, doi:10.1093/jxb/erv228.

8. Anžič, B. Transformation of periwinkle (*Catharanthus roseus*) for studying infection with '*Candidatus* Phytoplasma solani' and enzymatic activities in infectedon of Madagascar plants, University of Ljubljana, https://repozitorij.uni-lj.si/IzpisGradiva.php?id=111641&lang=slv, 2019.
